# Oral Care in Trauma Patients Admitted to the ICU: Viewpoints of ICU Nurses

**DOI:** 10.5812/traumamon.15110

**Published:** 2014-03-18

**Authors:** Seyed Alireza Javadinia, Zahra Kuchi, Alireza Saadatju, Mohsen Tabasi, Mohsen Adib-Hajbaghery

**Affiliations:** 1 Student Research Committee, Birjand University of Medical Sciences, Birjand, IR Iran; 2 Behavioral Sciences Research Center, Baqiyatallah University of Medical Sciences, Tehran, IR Iran; 3 Intensive Care Department, Imam Reza Hospital, Birjand University of Medical Sciences, Birjand, IR Iran; 4 Nursing and Midwifery Research Center, Birjand University of Medical Sciences, Birjand, IR Iran; 5 Student Research Committee, Pasteur Institute of Iran, Tehran, IR Iran; 6 Department of Medical-Surgical Nursing, Kashan University of Medical Sciences, Kashan, IR Iran

**Keywords:** Oral Hygiene, Respiration, Artificial, Wounds and Injuries, Intensive Care Units

## Abstract

**Background:**

Many patients with severe traumatic injuries are admitted to intensive care units (ICU). These patients usually require prolonged mechanical ventilation. These interventions require oral intubation and leave the mouth open which consequently impairs the natural antimicrobial activity in the mouth and airways. These patients are also prone to ventilator-associated pneumonia (VAP). Evidence shows that paying attention to oral hygiene in patients under mechanical ventilation is important in helping to prevent VAP.

**Objectives:**

The present study was conducted to assess the viewpoints and performance of ICU nurses at Birjand hospitals towards oral care of patients under mechanical ventilation.

**Patients and Methods:**

A cross-sectional study was conducted at ICUs of Imam-Reza and Vali-Asr hospitals, Birjand, Iran. Sampling was done through a census in which 53 ICU nurses participated. Descriptive statistics, Kolmogorov-Smirnov test, Mann-Whitney U tests and Kendall's correlation coefficient were used to analyze the data.

**Results:**

A total of 53 nurses participated in this study. Most of the nurses had been trained to provide oral care during their university education. According to the participants' opinions, oral care with an average score of 5.72 ranked second among the 10 nursing care domains. The most frequent oral care provided was oral suctioning, normal saline irrigation, and chlorhexidine rinse with 95%, 90%, and 81.3% frequency, respectively.

**Conclusions:**

Nurses participating in this study considered oral care to be of prime importance. Most of the participants although trained in this area felt the need for continuing training courses.

## 1. Background 

Trauma is one of the leading causes of morbidity and mortality worldwide (1). Providing care for patients with severe injuries is a challenging problem in critical care medicine. During the early stages of hospital care, trauma patients may require management in a variety of settings including the emergency department, radiology department, and operating room; many of them are finally admitted to the Intensive Care Unit (ICU). Once the severely injured patient has been transferred to the ICU, his\her management consists of the provision of high-tech quality care and implementation of strategies to stabilize the patient, optimize the hemodynamic status and oxygenation, provide good hygiene and prevent local and systemic complications (2). These patients usually require prolonged bladder and bowel care, minimum environmental stimulation, suitable positioning, maintenance of patient safety and mechanical ventilation (3). In some important life sustaining interventions such as mechanical ventilation and nutrition, the patient’s oral cavity is used as an important and easy entry for passing endotracheal and orogastric tubes. These interventions leave the patient's mouth open which consequently impairs the natural functions of the mouth. These patients are at risk for ventilator-associated pneumonia (VAP) (4). Studies have shown that VAP is common among trauma patients in ICUs and dramatically increases the mortality rate of these patients (5). VAP accounts for over 47% of all infections in ICUs (6), and 8% to 28% of patients under ventilators; VAP increases fatality by approximately 24% to 70% (7-9). It also leads to more prolonged use of ventilators, increases length of stay in the ICU as well as the costs of treatment (10). Evidence shows that monitoring oral hygiene in patients under the ventilator is one of the major routes to prevent VAP (11). Insufficient oral care leads to the colonization and aspiration of microbes that can lead to pneumonia (12). Insufficient oral care can also lead to a reduction of the salivary volume, dryness of the mouth, formation of dental plaque, gingival swelling, colonization of bacteria, stomatitis, dental infections and dental caries (13, 14). A study by Mori et al. showed that VAP rate can be decreased with regular oral care (11). Despite the importance of oral care in preventing VAP, insufficient studies have been published addressing this issue in Iran. Our review found only one study on the barriers of good oral care in ICUs from Iran. This study showed inadequate attention of nurses to this important aspect of care. In the aforementioned study, Adib-Hajbaghery et al. reported that only 29% of nurses were educated on oral care and the most commonly used method was mouth suctioning. Among the participants of the study, 20% did not take care of the patients’ mouth during their shifts; their overwhelming work load and lack of adequate personnel were the most important obstacles preventing provision of oral care (15). Also, in two review articles it was concluded that patients admitted to the ICU are at the greatest risk of acquiring VAP due to various reasons such as weak immune system , medication side effects, reduction of liquid intake, and existence of a tracheal tube in their mouth. Oral care is not done properly in ICUs and lack of a standard protocol for provision of such care is a contributing factor to this problem (9, 16). 

## 2. Objectives

Because of the importance of oral care in ICU patients under mechanical ventilation, and due to the paucity of studies conducted on performance of Iranian nurses in this regard, we found it necessary to investigate this issue in several medical centers. Therefore, the present study was conducted to assess the opinions and performance status of nurses working in ICUs of Birjand hospitals concerning rendering oral care to patients under mechanical ventilation.

## 3. Patients and Methods

This cross-sectional study was conducted in 2012 in ICUs of the Imam-Reza and Vali-Asr hospital, Birjand, Iran. Sampling was performed through the census method in which 53 nurses who were working in the mentioned departments participated. After approval of the proposal by the Research Committee of Birjand University of Medical Sciences and obtaining the necessary permits from the university and the hospitals, the researchers attended ICUs at the start of the working hours. Next, essential explanations were given to the nurses and after they agreed to participate, they were provided with a questionnaire. For data collection, a questionnaire was used where its validity and reliability was confirmed by a previous study (15). The questionnaire consisted of four parts. The first section included questions on demographic information (including age, gender, experience in the ICU, level of qualification, and history of oral care training). The second section consisted of eight questions. The first and second questions were about the frequency of oral assessment and oral care during the usual working shift. Questions three to five had yes or no replies and concerned the time of oral care in patient's chart, prior training on oral care, and using a checklist in oral care. The sixth question included a list of 10 nursing care procedures (including suctioning of trachea, skin care, eye care, intestinal function, writing reports, performing patients’ personal hygiene, taking care of catheters, nutrition, and preventing sensory overload). The participants were asked to rank these activities from one to ten. Giving a higher score to a question meant that greater priority was given to that issue. Question seven included a diagram of 12 columns relating to materials and tools used in oral care (such as cotton swabs, pieces of cotton gauze, normal saline, water, lime juice and glycerin, sodium hypochlorite, chlorhexidine, povidone iodine, tooth brush, tooth paste, and mouth suction). Participants were asked to mark each on a scale from one to 100 according to the frequency of usage. A higher number meant more usage of that item in oral care. Question number eight included a list of 10 factors which were known as obstacles in rendering oral care (including unpleasantness of oral care, lack of necessary tools for oral care, lack of time, insufficient number of nurses, not being as important as other types of care, lack of knowledge and skills in oral care, being afraid of the movements of tracheal tube and aspiration, and believing that this is not a nurse's duty). Participants were asked to give scores of one to ten to these factors and rank them according to their importance. Giving the same score to different items was not permitted.

### 3.1. Ethical Considerations

This study was approved by the institutional review board and the research ethics committee of Birjand University of Medical Sciences (Birjand, Iran). All subjects were informed about the voluntary nature of participation in the research and non-disclosure of personal information. They were briefed on the study aims and signed a written informed consent before taking part.

### 3.2. Data Analysis

Data analysis was performed using SPSS software version 16. Descriptive and analytical statistics were used. The distribution of data was examined using Kolmogorov-Smirnov test and nonparametric tests were applied due to the absence of normal distribution. Mann-Whitney U test was used to compare the mean oral care scores according to gender, education on oral care, number of times oral care was performed (less than twice and more than twice), age (less than 30 years and 30 and over) and work history (less than two years and two or more). Also, Kendall's correlation coefficient was used to examine the correlation between the oral care score, age and work records. A p-value of less than 0.05 was assumed to be significant. 

## 4. Results

**Figure 1.**
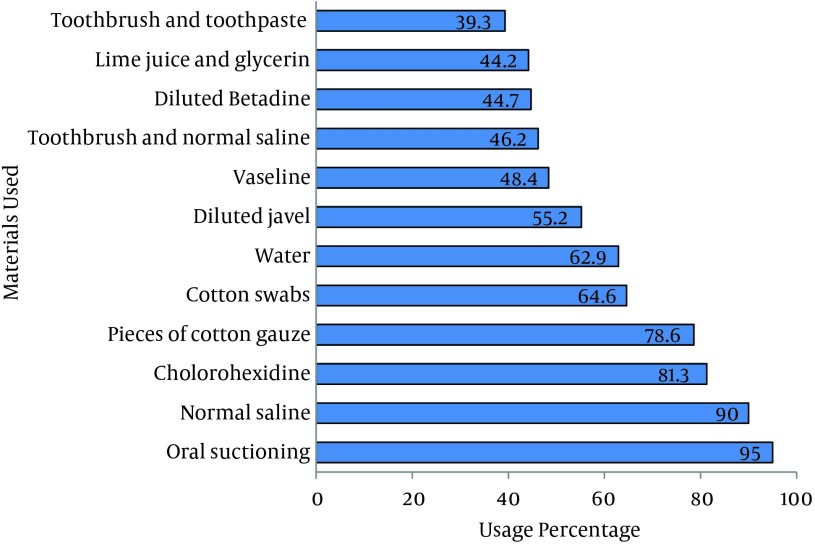
Frequency of Usage of Different Oral Care Materials and Instruments

**Figure 2.**
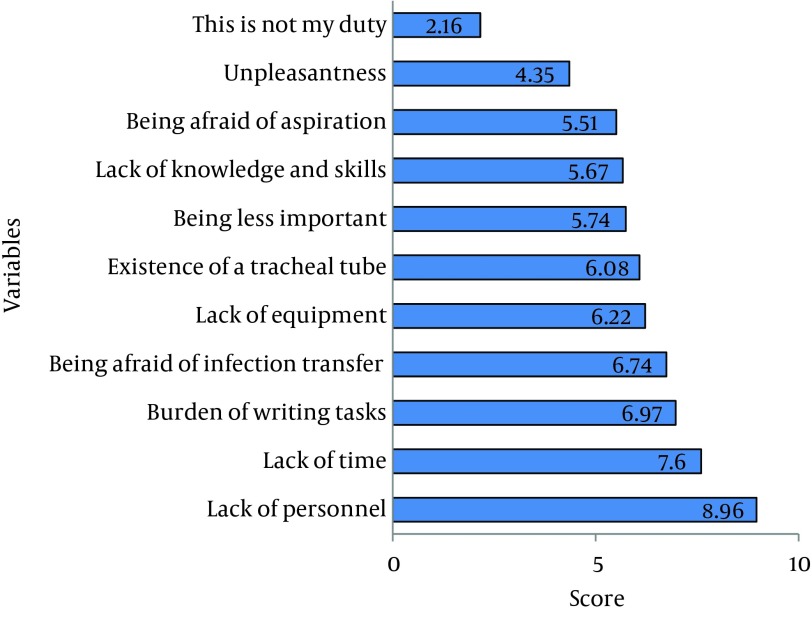
Ranking of Obstacles in Oral Care According to the Nurses

A total of 53 nurses participated in this study and 94.3% (50) were female. Also, 94.3% (50) had a Bachelor of Science degree. Nursing experience of most participants (54.7%, 29) was more than two years and their employment type was mostly through a contract (39.6%). Moreover, 32 nurses (60.4%) worked at Imam-Reza hospital (Table 1). Forty five participants (84.9%) were trained on oral care of patients under mechanical ventilation and most of the training was provided as part of their university training (62.3%, 33), re-training (6) or in the department (6). In terms of the frequency of oral care provided during each work shift, 20 nurses (37.7%) stated that they performed oral care twice daily. Only 15 nurses (28.3%) used a checklist for performing oral care and 62.3% (33) stated that they immediately recorded the care provisions in the patients' file (Table 2). In the participant’s opinion, oral care with a score of 5.72 was ranked second among the ten priorities of nursing care (Table 3). Mann-Whitney U test showed no significant differences between the mean oral care score according to gender (P = 0.28), age (P = 0.16), work experience (P = 0.19 ) and the level of education (P = 0.07). Also Kendall's correlation coefficient showed no correlation between the oral care score and age (r = 0.13, P = 0.19) or work experience (r = 0.07, P = 0.46). The most frequently used tools and materials of oral care were simple suctioning, normal saline swabs, and chlorhexidine that were used in 95%, 90%, and 81.3% of cases, respectively (Figure 1). Participants mentioned that lack of time and personnel were the most important factors preventing provision of proper oral care to patients under mechanical ventilation (Figure 2). 

**Table 1.** Demographic Information of the Participants

Variables	Frequency	Percentage
**Gender**		
Female	50	94.3
Male	3	5.7
**Age, y**		
< 30	32	60.4
> 30	21	39.6
**Level of education**		
Diploma	2	3.8
B.S.	50	94.3
M.S.	1	1.9
**Work history in the ICU, y**		
≤ 2	24	45.3
> 2	29	54.7
**Employment status**		
Permanent	17	32.1
Contract	21	39.6
Compulsory government services	15	28.3
**Hospital**		
Imam-Reza	32	60.4
Vali-asr	21	39.6

**Table 2.** Frequency of Care Rendered to Patients Under Mechanical Ventilation

	Frequency	Percentage
**Frequency of examining patient’s mouth during each shift**		
1 Time	6	11.3
2 Times	20	37.7
3 Times	19	35.8
4 Times	8	15.1
**Frequency of oral care provided during each shift for each patient**		
1 Time	7	13.2
2 Times	26	49.1
3 Times	16	30.2
4 Times	3	5.7
5 Times	1	1.9
**Using a special checklist for oral care**		
Yes	15	328.3
No	28	71.7
**Recording data after each session of oral care**		
Yes	33	62.3
No	20	37.7

**Table 3.** Mean and Standard Deviation of the Scores Given to Ten Nursing Care Procedures

Care type	The Mean Priority Score	Standard Deviation
**Tracheal suctioning**	9.02	1.83
**Mouth care**	5.72	1.42
**Skin care**	5.64	2.71
**Nutrition**	5.34	2.16
**Catheter care**	5.30	2.94
**Personal hygiene**	5.28	6.47
**Eye care**	4.98	3.15
**Preventing sensory overload **	4.91	3.20
**Report keeping**	4.58	3.21
**Intestinal function care**	4.34	2.68

## 5. Discussion

Nurses in this study considered oral care as their second most important priority after airway care. However, although the score of tracheal suctioning was 9.02, the average score given to oral care was considerably less. This score was not considerably different from scores given to skin care, nutrition care, catheter care, and providing personal hygiene. This outcome indicates that the importance of oral care is viewed as considerably lower than the importance of airway care. These results show the necessity for establishment of retraining courses in oral care for nurses. In a previous study, Adib-Hajbaghery et al. reported that nurses did not give a high score to oral care and this factor gained the seventh rank among ten nursing care priorities (15). Also in a study reported by Grap et al. 47% of the nurses thought that oral care is of little importance (17). However, Ganz et al. have reported that 44% of nurses questioned in their study gave high priority to oral care (12). Nonetheless, in most studies, the average priority score given to oral care was higher than the present study. This fact supports the need for retraining nurses in oral care and its role in preventing respiratory infections. About 85% of the participants in the present study reported that they were trained for oral care of patients under mechanical ventilation. This finding is in contrast with results of Adib-Hajbaghery et al. who reported that more than 70% of nurses had not been trained for oral care of patients under mechanical ventilation (15). In a study conducted on ICU nurses in England, 62% said that they had been trained in this field (18). Also a study on nurses of 59 ICUs in Europe showed that 77% of nurses had been trained in this regard (19). The results of these studies match the present study. However, although most of nurses in the present study had been trained on oral care, they did not give a high priority score to this aspect of care. This finding shows that nurses of ICUs need more professional re-training in this field. While most of the participants of this study reported that they recorded all cases of oral care in the patients charts, only 30% stated that they usually record oral care immediately after its provision. In the study reported by Adib-Hajbaghery et al. more than 86% of participants had stated that they usually record all cases of care in the patient charts; however, only 60% of oral care cases were recorded in the charts (15). Previous studies have shown that proper oral care and documentation of care provision could reduce the rate of VAP in ICUs (9, 16, 20). However, consistent with the findings of the present study, Grap et al. (17) and Hanneman et al. (21) reported that there is inconsistency between what nurses stated and what was recorded in the patient charts. Generally, the recorded oral care was less than what was performed. These findings show that nurses might ignore the importance of precise documentation; thus, more supervision is needed for precise performance and recording of oral care. Participants of this study mentioned that lack of time and personnel were the major reasons for ignoring oral care in patients under mechanical ventilation. This finding was consistent with Adib-Hajbaghery et al. who reported that burden of recording tasks and lack of time and personnel were the most important obstacles for oral care in ICUs (15). The study conducted in 59 ICUs in Europe also showed that one-third of nurses described oral care as unpleasant and two-thirds mentioned that it was difficult to perform (19). Other studies conducted on the barriers of quality care in different units such as the ICU have reported that factors such as lack of time, insufficient number of nurses, work burden, shortage of management and supervision, ignorance of managers towards the value of scientific practice, out-dated knowledge of nurses and unavailability of standard protocols for patient care were the obstacles of standard and evidence-based nursing care (19, 22, 23). The present study showed that only about 28% of the participants stated that they used a special checklist for examining and caring of the mouth. In a study by Adib-Hajbaghery et al. only 15% of nurses used a checklist for oral care (15). This is consistent with two other studies indicating that creating caring protocols within the re-training programs have been able to improve the nurse’s knowledge and clinical functioning (24, 25). Ganz et al. stated that lack of proper protocols is one of the major reasons for not performing oral care (12). Other studies have reported that there is no protocol for oral care in most European ICUs (26, 27). These results clarify the need to prepare a checklist and a special protocol for performing oral care in ICUs. The present study showed that mouth suctioning was the most common method used by nurses for oral care. This finding is consistent with the results of Adib-Hajbaghery et al. (15). These results do not match the most recent overseas studies which emphasize the use of tooth brushes in oral care of ICU patients for removing dental plaque and microbes (28). Although some studies have shown that mouth wash with chlorhexidine is more effective than tooth brushing in reduction of VAP (29), several studies have shown that mechanical methods such as tooth brushing are far better than swab and chlorhexidine (28-31). It has been reported that brushing twice a day is more effective in preventing pneumonia than using a swab (32). Using a tooth brush is only prohibited for a few cases such as patients with coagulation problems or extensive oral ulcers (33). In the present study, oral care of patients under mechanical ventilation was ranked second place, although its score was considerably lower than the score of airway care which obtained the first rank. Because of the close relationship between oral hygiene and the risk of pneumonia in these patients, these results show the need to hold re-training courses emphasizing the importance of oral care. It should be taken into consideration that the real performance (as opposed to the self-reported performance) of nurses in oral care was not observed here and it is suggested that further studies be conducted on performance of the caring team in oral care of patients under mechanical ventilation.

## References

[1] Richards CF, Mayberry JC (2004). Initial management of the trauma patient.. Critic Care Clin..

[2] Haddad SH, Arabi YM (2012). Critical care management of severe traumatic brain injury in adults.. Scand J Trauma Resusc Emerg Med..

[3] Ryan DL (2009). Caring for patients with traumatic brain injuries: Are you up to the challenges? .. Am Nurse Today ..

[4] Dale C, Angus JE, Sinuff T, Mykhalovskiy E (2013). Mouth care for orally intubated patients: a critical ethnographic review of the nursing literature.. Intensive Crit Care Nurs..

[5] Magnotti LJ, Croce MA, Fabian TC (2004). Is ventilator-associated pneumonia in trauma patients an epiphenomenon or a cause of death?. Surg Infect (Larchmt)..

[6] Cason CL, Tyner T, Saunders S, Broome L, Centers for Disease Control and Prevention . (2007). Nurses' implementation of guidelines for ventilator-associated pneumonia from the Centers for Disease Control and Prevention.. Am J Crit Care..

[7] Fagon JY, Chastre J, Domart Y, Trouillet JL, Pierre J, Darne C (1989). Nosocomial pneumonia in patients receiving continuous mechanical ventilation. Prospective analysis of 52 episodes with use of a protected specimen brush and quantitative culture techniques.. Am Rev Respir Dis..

[8] Chastre J, Fagon JY (2002). Ventilator-associated pneumonia.. Am J Respir Crit Care Med..

[9] Hajibagheri A, Azizi Fini I (2012). Mouth Care in Patients Receiving Mechanical Ventilation: A Systematic Review.. Nurs Midwifery Stud..

[10] Fagon JY, Chastre J, Hance AJ, Montravers P, Novara A, Gibert C (1993). Nosocomial pneumonia in ventilated patients: a cohort study evaluating attributable mortality and hospital stay.. Am J Med..

[11] Mori H, Hirasawa H, Oda S, Shiga H, Matsuda K, Nakamura M (2006). Oral care reduces incidence of ventilator-associated pneumonia in ICU populations.. Intensive Care Med..

[12] DeKeyser Ganz F, Fink NF, Raanan O, Asher M, Bruttin M, Nun MB (2009). ICU nurses' oral-care practices and the current best evidence.. J Nurs Scholarsh..

[13] Munro CL, Grap MJ (2004). Oral health and care in the intensive care unit: state of the science.. Am J Crit Care..

[14] Johnstone L, Spence D, Koziol-McClain J (2010). Oral hygiene care in the pediatric intensive care unit: practice recommendations.. Pediatr Nurs..

[15] Adib-Hajbaghery M, Ansari A, Azizi-Fini I (2013). Intensive care nurses' opinions and practice for oral care of mechanically ventilated patients.. Indian J Crit Care Med..

[16] Adib-Hajbaghery M, Ansari A, Azizi-Fini E (2011). [Oral care in ICU patients: a review of research evidence].. Feyz J Kashan Univ Med Sci..

[17] Grap MJ, Munro CL, Ashtiani B, Bryant S (2003). Oral care interventions in critical care: frequency and documentation.. Am J Crit Care..

[18] Jones H, Newton JT, Bower EJ (2004). A survey of the oral care practices of intensive care nurses.. Intensive Crit Care Nurs..

[19] Rello J, Koulenti D, Blot S, Sierra R, Diaz E, De Waele JJ (2007). Oral care practices in intensive care units: a survey of 59 European ICUs.. Intensive Care Med..

[20] Cutler CJ, Davis N (2005). Improving oral care in patients receiving mechanical ventilation.. Am J Crit Care..

[21] Hanneman SK, Gusick GM (2005). Frequency of oral care and positioning of patients in critical care: a replication study.. Am J Crit Care..

[22] Schwartz AJ, Powell S (2009). Brush up on oral assessment and care.. Nursing..

[23] Allen Furr L, Binkley CJ, McCurren C, Carrico R (2004). Factors affecting quality of oral care in intensive care units.. J Adv Nurs..

[24] Garcia R, Jendresky L, Colbert L, Bailey A, Zaman M, Majumder M (2009). Reducing ventilator-associated pneumonia through advanced oral-dental care: a 48-month study.. Am J Crit Care..

[25] Tolentino-DelosReyes AF, Ruppert SD, Shiao SY (2007). Evidence-based practice: use of the ventilator bundle to prevent ventilator-associated pneumonia.. Am J Crit Care..

[26] Sole ML, Byers JF, Ludy JE, Zhang Y, Banta CM, Brummel K (2003). A multisite survey of suctioning techniques and airway management practices.. Am J Crit Care..

[27] Feider LL, Mitchell P (2009). Validity and reliability of an oral care practice survey for the orally intubated adult critically ill patient.. Nurs Res..

[28] Pedreira ML, Kusahara DM, de Carvalho WB, Nunez SC, Peterlini MA (2009). Oral care interventions and oropharyngeal colonization in children receiving mechanical ventilation.. Am J Crit Care..

[29] Munro CL, Grap MJ, Jones DJ, McClish DK, Sessler CN (2009). Chlorhexidine, toothbrushing, and preventing ventilator-associated pneumonia in critically ill adults.. Am J Crit Care..

[30] Berry AM, Davidson PM (2006). Beyond comfort: oral hygiene as a critical nursing activity in the intensive care unit.. Intensive Crit Care Nurs..

[31] Panchabhai TS, Dangayach NS, Krishnan A, Kothari VM, Karnad DR (2009). Oropharyngeal cleansing with 0.2% chlorhexidine for prevention of nosocomial pneumonia in critically ill patients: an open-label randomized trial with 0.01% potassium permanganate as control.. Chest..

[32] Binkley C, Furr LA, Carrico R, McCurren C (2004). Survey of oral care practices in US intensive care units.. Am J Infect Control..

[33] Abidia RF (2007). Oral care in the intensive care unit: a review.. J Contemp Dent Pract..

